# Dietary Exposure to Essential and Non-essential Elements During Infants’ First Year of Life in the New Hampshire Birth Cohort Study

**DOI:** 10.1007/s12403-022-00489-x

**Published:** 2022-06-16

**Authors:** Antonio J. Signes-Pastor, Vicki Sayarath, Brian Jackson, Kathryn L. Cottingham, Tracy Punshon, Margaret R. Karagas

**Affiliations:** 1 Department of Epidemiology, Geisel Medical School at Dartmouth College, Lebanon, NH, USA; 2 Instituto de Investigación Sanitaria y Biomédica de Alicante, Universidad Miguel Hernández (ISABIAL-UMH), Alicante, Spain; 3 CIBER de Epidemiología y Salud Pública (CIBERESP), Instituto de Salud Carlos III (ISCIII), 28029 Madrid, Spain; 4 Department of Earth Sciences, Dartmouth College, Hanover, NH, USA; 5 Department of Biological Sciences, Dartmouth College, Hanover, NH, USA

**Keywords:** Mixture, Essential elements, Non-essential elements, Food, Biomarkers of exposure

## Abstract

Even the low levels of non-essential elements exposure common in the US may have health consequences especially early in life. However, little is known about the infant’s dynamic exposure to essential and non-essential elements. This study aims to evaluate exposure to essential and non-essential elements during infants’ first year of life and to explore the association between the exposure and rice consumption. Paired urine samples from infants enrolled in the New Hampshire Birth Cohort Study (NHBCS) were collected at approximately 6 weeks (exclusively breastfed) and at 1 year of age after weaning (*n* = 187). A further independent subgroup of NHBCS infants with details about rice consumption at 1 year of age also was included (*n* = 147). Urinary concentrations of 8 essential (Co, Cr, Cu, Fe, Mn, Mo, Ni, and Se) and 9 non-essential (Al, As, Cd, Hg, Pb, Sb, Sn, V, and U) elements were determined as a measure of exposure. Several essential (Co, Fe, Mo, Ni, and Se) and non-essential (Al, As, Cd, Hg, Pb, Sb, Sn, and V) elements had higher concentrations at 1 year than at 6 weeks of age. The highest increases were for urinary As and Mo with median concentrations of 0.20 and 1.02 μg/L at 6 weeks and 2.31 and 45.36 μg/L at 1 year of age, respectively. At 1 year of age, As and Mo urine concentrations were related to rice consumption. Further efforts are necessary to minimize exposure to non-essential elements while retaining essential elements to protect and promote children’s health.

## Introduction

Exposure to non-essential elements such as arsenic (As), lead (Pb), mercury (Hg), and cadmium (Cd) has become a significant global health issue owing to their frequency and toxic effects on human health (ATSDR 2019a). This concern is particularly relevant for infants and young children for whom non-essential element exposures, even at the low levels common in the United States of America (US) and elsewhere, may have health consequences (Farzan et al. 2016; Nadeau et al. 2014; Vahter et al. 2020; Wasserman et al. 2014).

There is a growing body of evidence reporting high levels of non-essential elements in foods for infants and young children (Arcella et al. 2021; EFSA 2009a; Karagas et al. 2016; Signes-Pastor et al. 2016), which supports that food intake is a source of essential but also non-essential elements (FDA 2020a). The US Subcommittee on Economic and Consumer Policy of the Committee on Oversight and Reform of the House of Representatives reported that US baby foods have levels of As, Pb, Cd, and Hg higher than current standards for food or water (Congress 2021a, b). Consumption of rice and rice containing foods are common in infants’ and young children’s food (e.g., during weaning) because of its putative organoleptic and nutritional value and relatively low allergenic potential. However, consumption of rice and rice-based products relate to an increase of urinary As concentrations (Davis et al. 2017; Karagas et al. 2016; Signes-Pastor et al. 2018). Other non-essential elements, such as Cd and Pb, are also accumulated in foods grown with contaminated soil and water; the contamination comes from both natural and anthropogenic sources, such as agricultural and industrial activities (EFSA 2009b, 2010). Regulations to set maximum allowable levels of non-essential elements in food have recently been proposed or established to decrease exposure (EC 2015, 2021a, 2021b, 2021; FDA 2020b). Yet, further efforts are necessary to successfully minimize early-life toxic dietary exposures to protect public health (Congress 2021a; Nachman et al. 2018).

Very little data exist on biomarker measurements as internal exposures and exposure trends in essential and non-essential elements during the first year of life (Carignan et al. 2016; Karagas et al. 2016; Ljung et al. 2011; Signes-Pastor et al. 2017). Humans excrete several elements in urine after exposure and thus exposures can be assessed via urinary element concentrations (Fort et al. 2014). However, the rate of excretion in urine of each element may differ (ATSDR 2007, 2012a, 2010; EFSA 2009a, b; Vacchi-Suzzi et al. 2016).

In this study, we hypothesized that urinary element concentrations, as an indicator of internal exposure to essential [i.e., cobalt (Co), chromium (Cr), copper (Cu), iron (Fe), manganese (Mn), molybdenum (Mo), nickel (Ni), and selenium (Se)] and non-essential [i.e., aluminum (Al), As, Cd, Hg, Pb, antimony (Sb), tin (Sn), vanadium (V), and uranium (U)] elements would increase in the first year of life following the introduction of foods other than breastmilk, including solid foods among previously exclusively breastfed infants. To test our hypothesis, we assessed element concentrations in urine samples collected at 6 weeks of age before weaning and approximately 1 year of age among the same infants. Moreover, based on our prior findings on rice consumption and As exposure early in life (Karagas et al. 2016), we also investigated the associations between rice and rice-based products consumption and the concentrations of other non-essential and essential elements in urine samples from one-year-old infants.

## Methods

### Study Population

Our study comprised infants enrolled in the New Hampshire Birth Cohort Study (NHBCS), a longitudinal pregnancy cohort designed to examine the impacts of toxicants in drinking water and diet on maternal–child health. Since 2009, the NHBCS has recruited pregnant women 18–45 years of age at approximately 24–28 weeks of gestation from prenatal clinics in the rural state of New Hampshire. Eligibility criteria include English literacy, the use of a private, unregulated water system at home (e.g., private well), not planning to move during pregnancy, and a singleton birth as described previously (Gilbert-Diamond et al. 2011). Women were asked to complete a self-administered lifestyle and medical history questionnaire (Gilbert-Diamond et al. 2011; Karagas et al. 2016).

The Committee for the Protection of Human Subjects at Dartmouth College approved the study, and all participants provided written informed consent.

### Urine and Food Diary Collection

Spot urine samples were collected at approximately 6 weeks and 1 year of age in cotton urine pads and stored in polyethylene sterile containers. Samples were aliquoted into 1.8 ml vials within 24–72 h and frozen at −80 °C until analysis (Carignan et al. 2015).

The urine samples collection took place after completing a 3-day food diary. Infants’ parents or caregivers were asked to complete the food diary at the end of each day. The unstructured food diary included details of infants’ food and beverage intake during 3 consecutive days (e.g., time of feeding and type and amount of foods/beverage consumed). The food diaries were collected on paper during clinical visits (Carignan et al. 2015; Karagas et al. 2016; Signes-Pastor et al. 2018).

### Laboratory Analysis

We determined urinary concentrations of essential elements (i.e., Co, Cr, Cu, Fe, Mn, Mo, Ni, and Se) and non-essential elements (i.e., Al, As, Cd, Hg, Pb, Sb, Sn, U, and V) at the Trace Element Analysis Core at Dartmouth College. Urinary specific gravity was measured with a handheld refractometer with automatic temperature compensation (PAL-10S; ATAGO Co Ltd).

Elemental analysis of urine was conducted with an Agilent 8900 inductively coupled plasma-mass spectrometry (ICP-MS) in direct solution acquisition mode. Urinary As species concentrations were determined using the Agilent 8900 ICP-MS interfaced with an Agilent liquid chromatograph 1260 equipped with a Thermo AS7, 2 × 250-mm column, and a Thermo AG7, 2 × 50-mm guard column (Jackson 2015; Signes-Pastor et al. 2020).

Several NIST human urine standard reference materials 2669 level I and level II were analyzed in each analysis batch. The average (standard deviation) recoveries across batches (*n* = 3) for arsenobetaine, DMA, MMA, and iAs were 105% (3), 115% (9), 100% (4), and 101% (11), respectively. The limit of detection (LOD) was calculated as the mean of the blank concentrations plus 3 times their standard deviation multiplied by the dilution factor. The average LOD across analysis batches for each essential and non-essential element of interest in this study is reported in Tables S1 and S2. Only when the ICP-MS standard calibration curve provided zero or negative values the value of LOD/√2 was imputed (Lubin et al. 2004). The remaining urine concentrations, even those below the LOD, were not imputed, taking advantage of the ICP-MS wide linear dynamic range (EFSA 2009a). Missing values were assumed to be at random. The Multivariate Imputation by Chained Equations (MICE) method was applied to impute the missing values with the average values obtained from 5 generated complete datasets (Buuren 2011).

### Statistical Analysis

The urinary element concentrations, including the sum of urinary As species (ΣAs = inorganic arsenic + monomethylarsonic acid (MMA) + dimethylarsinic acid (DMA)), were divided by the specific gravity to correct for urine dilution (Nermell et al. 2008). The concentrations were positively skewed and thus they were natural logarithm transformed (Ln) before statistical analysis.

Our study population comprises 2 separately drawn subgroups from the NHBCS according to the availability of element concentrations in paired urine samples at 6 weeks and 1 year of age and one-year-old infants’ consumption of rice and rice-based products.

Subgroup 1 was used to evaluate changes in urinary elements from 6 weeks to 1 year of age. Subgroup 1 contained 187 infants exclusively breastfed at 6 weeks of age with paired urine samples at 6 weeks and 1 year of age analyzed for essential and non-essential element concentrations; 82 infants with missing dietary information at 6 weeks of age; and 79 consumers of formula or solid food at 6 weeks of age were excluded (Fig. S1A). The urinary Al and Sn concentrations contained 43 missing values each, which were imputed using MICE (Buuren 2011). In this subgroup, dietary information on rice consumption at 1 year of age was not available. The subgroup 1 dietary information was used to identify exclusively breastfed infants at 6 weeks consuming solid food at 1 year of age. The dietary information and urine samples were collected in 2014–19.

Subgroup 2 was used to evaluate the association between rice and rice product intake and urinary elements. Subgroup 2 contained 147 one-year-old infants with information on rice consumption after excluding 5 infants without urinary essential and non-essential elements data (Fig. S1B) (Karagas et al. 2016). In this subgroup, urinary Al, Sn, and Hg concentrations were excluded owing to the high proportion of imputed values (> 60%). The subgroup 2 dietary information regarding rice and rice-based product consumption and urine samples at 1 year of age were gathered in 2013–2014.

Using the infant study population subgroup 1, we assessed the urinary essential and non-essential element concentrations in samples collected at 6 weeks and 1 year of age descriptively and by performing paired *t* test analyses. We calculated the ratio between the concentrations at 1 year of age versus 6 weeks of age in the paired samples (i.e., $\frac{1\;\text{year}}{6\;\text{weeks}}$ urine concentrations) to explore magnitude of change in the urinary essential and non-essential element concentrations, as shown in Fig. S2. A ratio equal to 1 indicates that the concentrations did not change. We also performed the mixture approach Weighted Quantile Sum (WQS) regression using the assessment time point (i.e., 6 weeks vs. 1 year—binary) as the dependent variable. The WQS regression model included 40% of the dataset for training and 60% for validation, and 100 bootstrap samples for parameter estimation were assigned. The estimates of mixture effects and indicators of exposure importance (i.e., weights) were calculated with the WQS regression model by combining the exposures to an empirically weighted index (Carrico et al. 2015).

Using the infant study population subgroup 2, we evaluated urinary essential and non-essential element concentrations at 1 year of age in association with rice consumption within the 2 days prior to urine sample collection. Descriptive and two-sample *t* test analyses comparing rice consumers vs. non-rice consumers were performed.

## Results

Both infant study population subgroups had a slightly uneven distribution of boys and girls (45%/55% and 56%/44% of boys/girls in subgroup 1 and 2, respectively). Mothers were generally married (> 90%), and about 80% of them had a college graduate or any postgraduate schooling (Table 1).

**Fig. 1:**
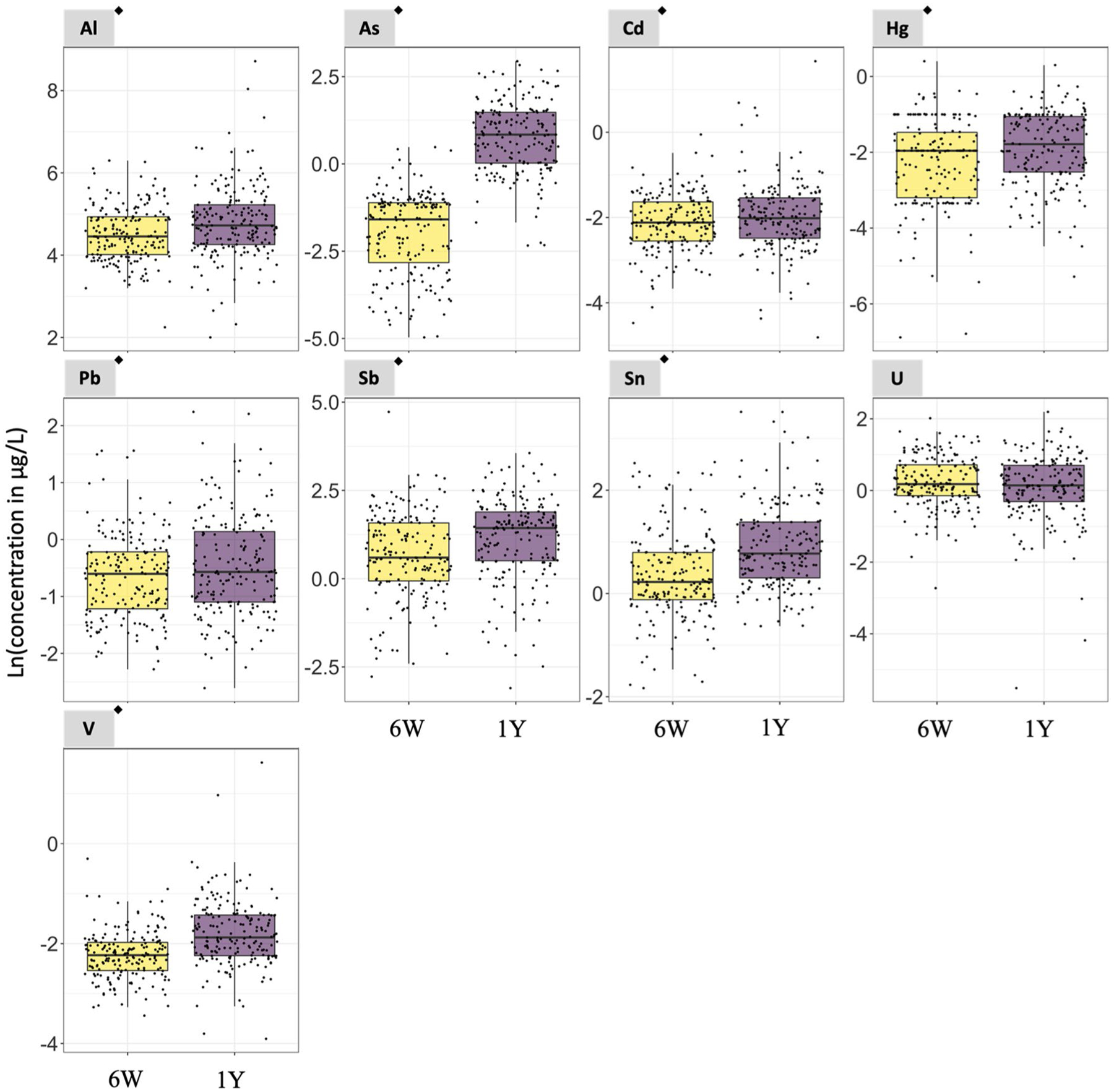
Urinary non-essential element concentrations in urine samples collected at 6 weeks and 1 year of age from the same set of infants. *N* = 187. ^◆^Statistically significant paired t test (*p*-value < 0.05. Table S3). 6W = 6 weeks of age. 1Y = 1 year of age. Notice that the scale of the y-axis varies to facilitate the visualization of the concentrations in each plot. The As concentrations refer to the sum of inorganic arsenic, monomethylarsonic acid, and dimethylarsinic acid

**Fig. 2:**
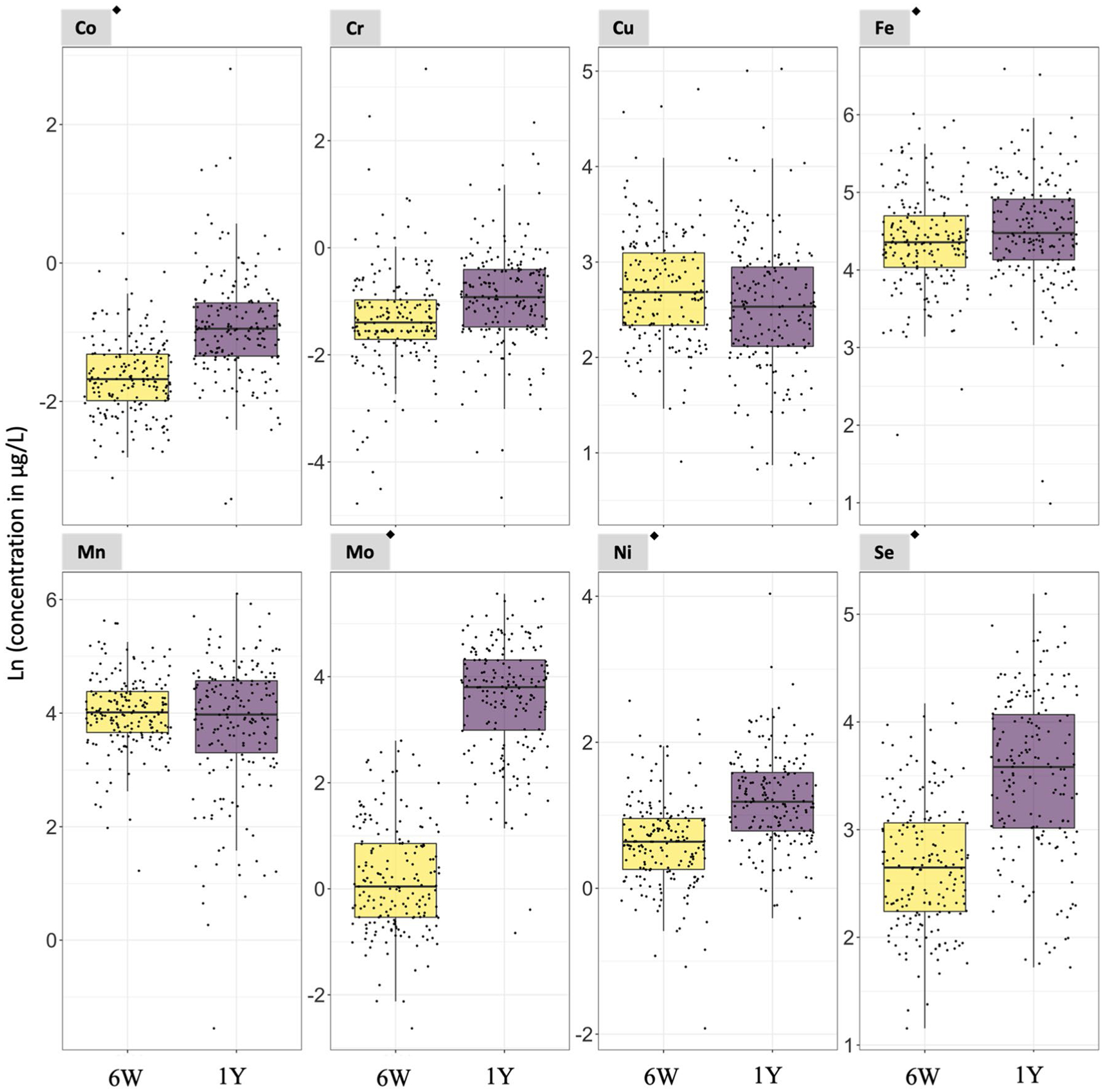
Urinary essential element concentrations in urine samples collected at 6 weeks and 1 year of age from the same set of infants. *N* = 187. ^◆^Statistically significant paired t test (*p*-value < 0.05. Table S3). 6W = 6 weeks of age. 1Y = 1 year of age. Notice that the scale of the y-axis varies to facilitate the visualization of the concentrations in each plot

In Subgroup 1, the concentrations of 8 of the 9 non-essential elements evaluated in the paired urine samples were higher at 1 year compared to 6 weeks of age (i.e., Al, ∑As, Cd, Hg, Pb, Sb, Sn, and V) with a *p*-value < 0.05 in paired t test analyses (Fig. 1 and Table S3). The urinary median concentrations of the non-essential elements at 6 weeks/1 year of age were 86.09/113.66 μg/L (Al), 0.20/2.31 μg/L (∑As), 0.12/0.13 μg/L (Cd), 0.14/0.17 μg/L (Hg), 0.55/0.57 μg/L (Pb), 1.82/4.20 μg/L (Sb), 1.25/2.17 μg/L (Sn), and 0.11/0.15 μg/L (V) (Table S3). In addition, 5 of the 8 essential elements had urinary concentrations higher at 1 year than at 6 weeks of age (i.e., Co, Fe, Mo, Ni, and Se) with a *p*-value < 0.05 in paired *t* test analyses (Fig. 2 and Table S3). The urinary median concentrations of the essential elements at 6 weeks/1 year of age were 0.19/0.39 μg/L (Co), 78.36/89.82 μg/L (Fe), 1.05/45.36 μg/L (Mo), 1.80/3.29 μg/L (Ni), and 14.14/36.39 μg/L (Se) (Table S3).

Among non-essential elements, the median ratio between concentrations at 1 year and 6 weeks of age concentrations ranged from 1.0 for U (i.e., no changes) to 14.8 for ∑As (i.e., a nearly 15-fold increase) (Table S3). The median ratios for Al, Cd, Hg, Pb, Sb, Sn, and V ranged from 1.1 to 1.7 (Table S3). Among essential elements, the median ratio between 1 year and 6 weeks of age ranged from 0.9 for Mn to 31.8 for Mo. The median ratios for Co, Cr, Cu, Fe, Ni, and Se ranged from 0.9 to 1.9 (Table S3). The distribution of $\frac{1\;\text{year}}{6\;\text{weeks}}$ of age natural logarithm-transformed (Ln) urinary essential and non-essential element concentrations are shown in Fig. S2.

The overall analysis of exposure to the element mixture at 1 year of age versus 6 weeks of age using WQS model regression assigned the highest positive weights to urinary ∑As (i.e., 0.516) and Mo (i.e., 0.289) concentrations followed by urinary Co with a weight 0.081 (Fig. S3). Urinary ∑As, Mo, and Co represented 51.6%, 26.9%, and 8.1% of the total weights of the mixture. For Hg, V, Sb, Ni, Cr, and U, the positive weights ranged from 0.001 to 0.028 with a percentage contribution to the total weights of the mixture ranging from 0.1 to 2.8% (Fig. S3). The remaining elements had weighted index close to zero. The WQS model regression did not identify any negative weights.

**Fig. 3:**
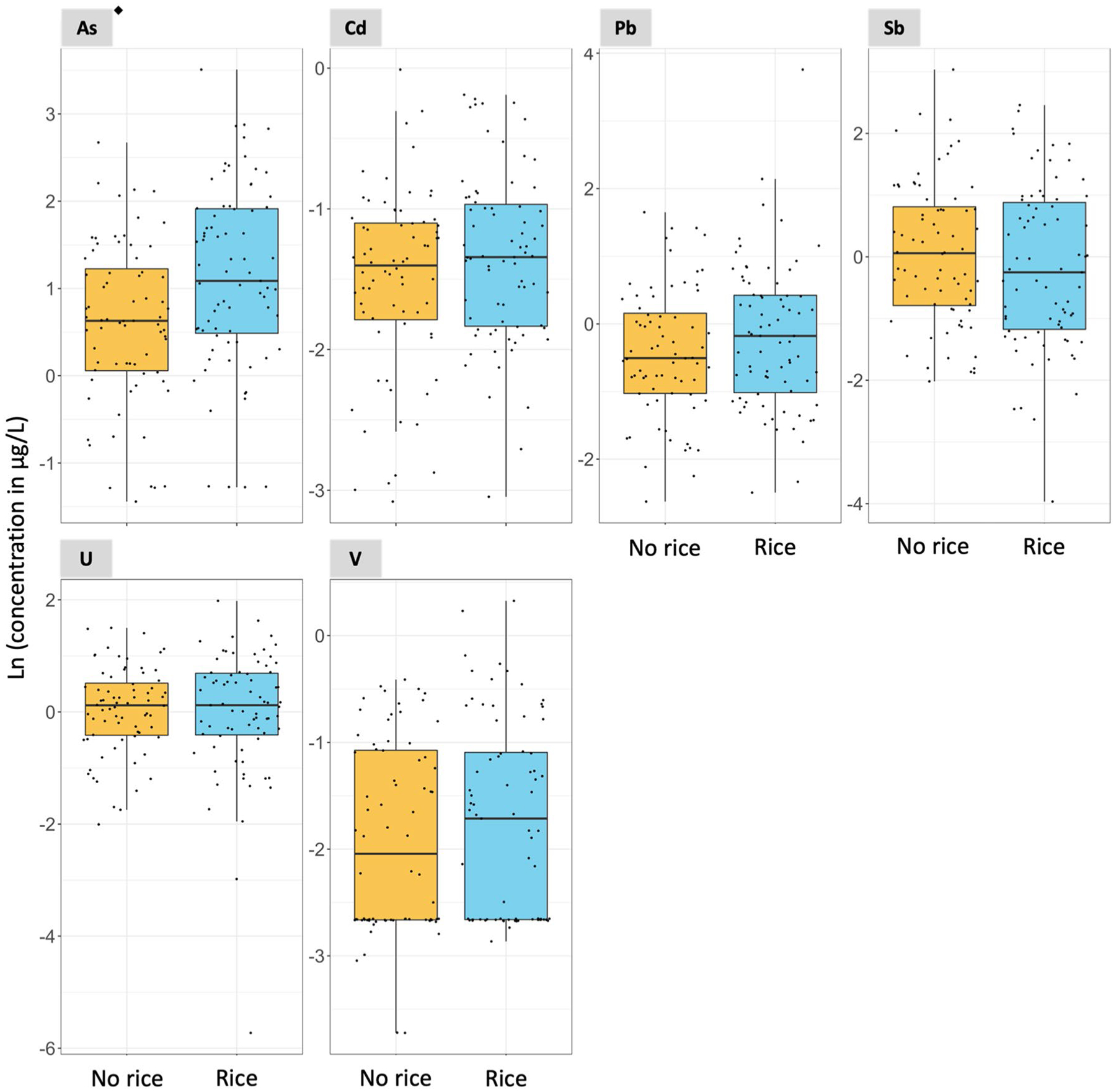
Association between urinary non-essential element concentrations and rice consumption at 1 year of age. *N* = 147. ^◆^Statistically significant two-sample t test (*p*-value < 0.05. Table S4). Notice that the scale of the y-axis varies to facilitate the visualization of the concentrations in each plot. The As concentrations refer to the sum of inorganic arsenic, monomethylarsonic acid, and dimethylarsinic acid

**Fig. 4:**
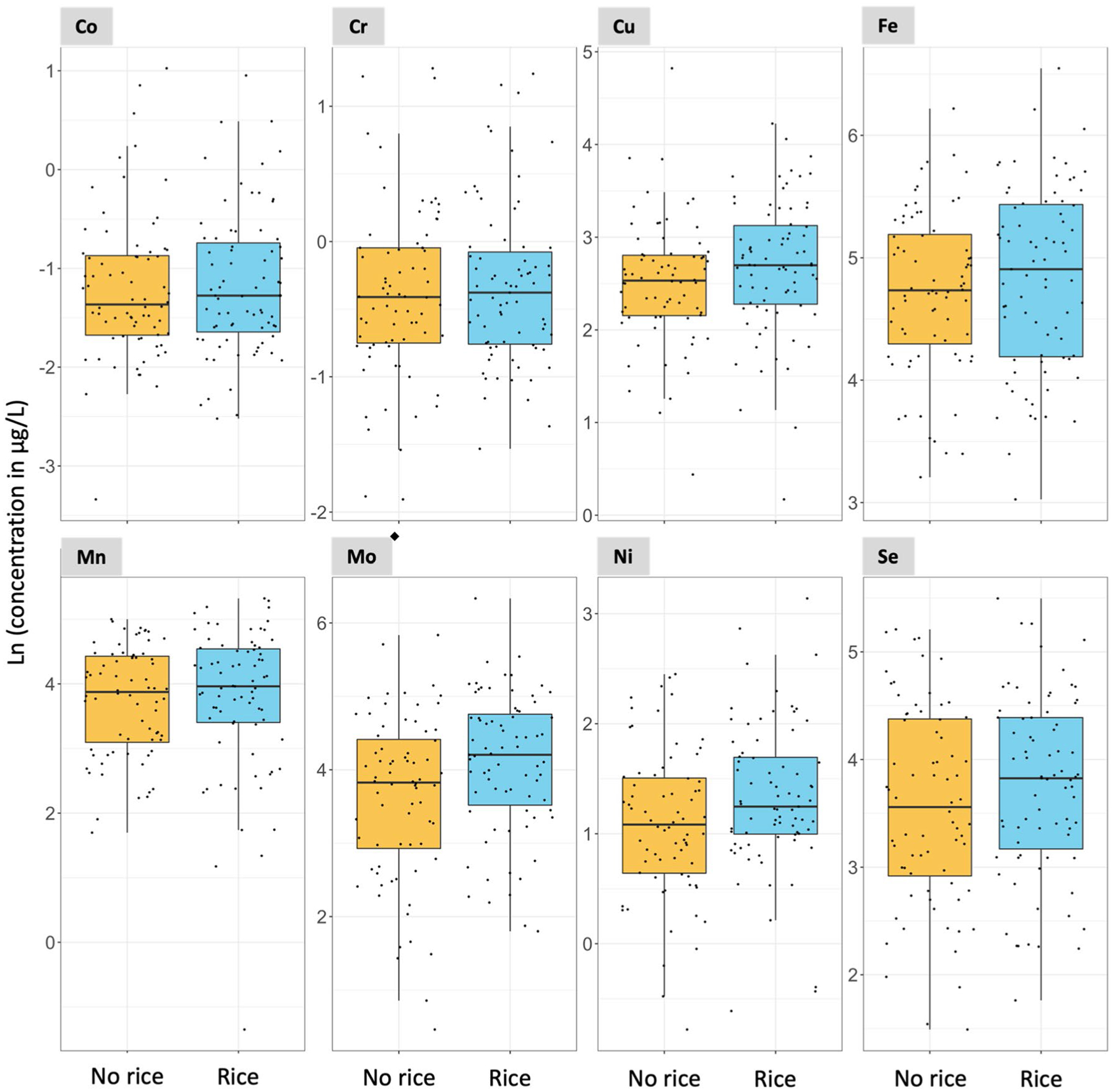
Association between urinary essential element concentrations and rice consumption at 1 year of age. *N* = 147. ^◆^Statistically significant two-sample t test (*p*-value < 0.05. Table S4). Notice that the scale of the y-axis varies to facilitate the visualization of the concentrations in each plot

In Subgroup 2, the consumption of rice at 1 year of age was associated with increased urinary ∑As and Mo concentrations with a *p*-value < 0.05 in two-samples *t* test analyses (Figs. 3, 4, and Table S4). The medians urinary ∑As were 2.96 and 1.88 μg/L for rice and no rice consumers, respectively. The medians urinary Mo concentrations were 67.01 and 45.90 μg/L for rice and no rice consumers, respectively (Table S4). Although urinary ∑As and Mo concentrations were only weakly correlated at 6 weeks of age (Spearman’s ρ = 0.18, Fig. S4), they were moderately correlated at 1 year of age (ρ = 0.64) (Fig. S5), among both infant rice (ρ = 0.48) (Fig. S6) and non-rice consumers (ρ = 0.55) (Fig. S7). The consumption of rice at 1 year of age was also associated with a borderline statistically significant increase in urinary Ni with a *p*-value of 0.053 in the two-sample *t* test analysis (Table S4).

## Discussion

In our US-based exclusively breastfed infant study population, we found increased urinary concentrations of non-essential (i.e., Al, As, Cd, Hg, Pb, Sb, Sn, and V) and essential elements (i.e., Co, Fe, Mo, Ni, and Se) at 1 year compared to 6 weeks of age. Among 1-year-old infants, urinary ∑As and Mo concentrations were higher for infants who consumed rice and rice-based products.

Inorganic arsenic is a well-known human carcinogen with increasing evidence that early-life exposure may increase the risk of a wide range of detrimental health effects (i.e., neurological, cardiovascular, respiratory, and metabolic diseases) with impacts throughout the life course (Farzan et al. 2016; IARC 2012; Rodríguez-Barranco et al. 2016; Signes-Pastor et al. 2019, 2021). We observed a median increase in infants’ urinary ∑As concentrations of 15-fold at 1 year compared to that at 6 weeks of age, concentrations at 1 year of age correlated with consumption of rice and rice-based products. The 1-year-old infants’ urinary ∑As are in line with earlier studies with an increased ∑As exposure during weaning in infants 6 to 9 month of age in the US with a median (range) of 0.99 (0.17–11.95) μg/L (Signes-Pastor et al. 2018) and in the United Kingdom (UK) of 2.81 (0.18–12.89) μg/L (Signes-Pastor et al. 2017).

Rice may contain higher As than other cereals and vegetables (Signes-Pastor et al. 2008; Williams et al. 2007). To reduce inorganic arsenic exposure, the maximum level of 100 μg/kg has been enforced for rice destined to produce foods for infants and young children in Europe (EC 2015). In the US, the 100 μg/kg of inorganic arsenic level in infant rice cereals is an action level but not a regulation (FDA 2020b), which could limit manufacturer compliance (Carey et al. 2018; Congress 2021a; FDA 2020a). Our study includes data gathered before the FDA action level was finalized in August 2020 (FDA 2020a), thus further studies will need to evaluate more recent exposures.

Besides inorganic arsenic, exposure to Cd, Hg, and Pb is also of public health concern. Cadmium is a human carcinogen, and Pb and Hg are strong neurotoxicants (ATSDR 1999, 2007, 2012a; EFSA 2015, 2010, 2009b). There is no defined safe level of exposure to inorganic arsenic, Cd, Hg, or Pb, yet detectable levels are being reported in baby foods (Brody and Houlihan 2019; Congress 2021a, b). This may explain the increased exposure to these non-essential elements in our infant study population between 6 weeks and 1 year of age. The current FDA plan, *Closer to Zero*, aims to reduce infants’ and young children’s exposure to toxic elements from food, but the effectiveness of the plan still needs to be evaluated (FDA 2021). Likewise, the European Commission has recently enforced stricter regulations regarding maximum limits of Cd and Pb in a wide variety of foods to reduce exposure (EC 2021a, b).

Of the other non-essential elements, ingestion of Al, Sb, Sn, and V from diet is among the primary exposure routes for non-occupationally exposed adults (ATSDR 2005a, 2008, 2012b, 2008; b; EFSA 2005). Consistent with this, we observed increased urinary concentrations in our one-year-old infants from 6 weeks of age. Aluminum is also associated with neurotoxicity (Dórea and Marques 2010). The median urinary Al concentration of 113.6 μg/L in our one-year-old infants was slightly higher than the upper bound reference value in urine for adults of 110 μg/L (Caroli et al. 1994; EFSA 2008) and thus warrants further investigation. At 1 year of age, the urinary levels of Sb, Sn, and V were each relatively low (ATSDR 2012b; b; Poddalgoda et al. 2016), and the levels of the essential elements Co, Fe, and Se reached similar levels to those reported in the general population (ATSDR 2003, 2004; Bresson et al. 2015; Pfrimer et al. 2014). The median urinary Ni concentration of 3.29 μg/L at 1 year of age was higher than the upper bound reference value of 3 μg/L for healthy adults (ATSDR, 2005b). Further studies are necessary to assess the health impact of the overall real-life simultaneous exposures to essential and non-essential elements (ATSDR 2012b; b; Poddalgoda et al. 2016).

In our mixture exposure assessment using WQS regression, the highest positive weights were assigned to urinary ∑As and Mo concentrations, suggesting that they are the largest contributors of the exposure mixture of essential and non-essential elements during weaning. The joint effect of an exposure mixture of inorganic arsenic and Mo on children’s health is still scarce (García-Villarino et al. 2021); however, both have been related to an increased oxidative stress (Domingo-Relloso et al. 2019; Tolins et al. 2014).

Urinary Mo concentrations were related to rice and rice product consumption among one-year-old infants. Rice is a source of the essential element Mo (Huang et al. 2019), and urine is the dominant excretion route for Mo (ATSDR 2020). This may explain the increased urinary Mo with rice consumption. Ingestion of Mo is a cofactor for important enzymes, such as aldehyde oxidase, xanthine dehydrogenase, sulfite oxidase, and amidoxime reducing component (Huang et al. 2019). The urinary Mo concentrations in our one-year-old infants were similar to the urinary Mo concentrations reported in a prior study of 496 US residents including both urban and rural communities, both males and females, and persons aged 6–88 years from all major ethnicities with a median (interquartile range: Q1–Q3) of 56.5 (27.9–93.9) μg/L (ATSDR 2020; Paschal et al. 1998). Rice can also accumulate Ni (Cao et al. 2017), which may also explain the higher urinary concentration trend among rice consumers compared to non-rice consumers. Among rice consumers, the median urinary Ni concentration (3.48 μg/L) was higher than the upper bound reference value for healthy adults (ATSDR 2005b).

While our findings are based on a modest sample size from a well-characterized cohort study, we nevertheless observed statistically significant increases in urinary concentrations of several essential and non-essential elements during their first year of life. However, the potential contribution of metabolic changes in the kinetics and excretion of essential and non-essential elements during children’s first year of life still needs to be explored (Skröder Löveborn et al. 2016). Urinary multi-element analysis using mass spectrometry was performed following established protocols (Pirkle 2012). In addition, we also performed urinary As speciation and calculated the summation of inorganic arsenic, MMA, and DMA (∑As) excluding non-toxic organoarsenical compounds (i.e., arsenobetaine) as a proxy for inorganic arsenic exposure, which allowed us to control for As exposure misclassification from unmetabolized forms (Jones et al. 2016). We used rice and rice-based products data from a food diary completed just before a spot urine sample collection, where element concentrations were determined as an internal exposure biomarker. This approach allowed us to capture rapidly excreted essential and non-essential elements in urine, such as As and Mo (ATSDR 2020; Meharg et al. 2014); however, urinary concentrations may not provide an accurate measurement of recent exposure for elements slowly released in the urine, such as Co and Cd (ATSDR 2004, 2012a). For the latter, urinary concentrations are a biomarker of long-term exposure (Vacchi-Suzzi et al. 2016). It is also important to bear in mind that the dietary information gathered with a food diary based on 3 consecutive days might not represent children’s typical food consumption pattern.

Information regarding biomarker concentrations of essential and non-essential elements among infants over their first year of life is scant, and despite concerns regarding non-essential elements in foods marketed for infants, limited data exist on whether such foods increase infant biomarker concentrations. Yet infancy is a crucial period of development and a time when sensitivity to toxicants may be greatest. Future efforts should aim to reduce toxic dietary exposures while preserving beneficial nutrients in foods consumed by infants and young children.

## Supplementary Material

Supplemental Material

## Figures and Tables

**Table 1:** Selected characteristics of study mothers and infants

Variables	Study sample for 6 weeks *vs*. 1 year of age infants’ urine comparisons (*n* = 187^#^)	Study sample for 1 year of age infants’ urine *vs*. rice consumption comparisons (*n* = 147*)
Gestational age (weeks)	39.29 (33.86, 38.56–40.14, 42.29)	39.14 (31.43, 38.43–40.00, 41.86)
Maternal pre-pregnancy BMI	23.40 (16.82, 23.40–26–37, 41.75)	24.03 (17.37, 21.50–27.96, 48.18)
Maternal education		
< 11th grade or high school graduate or equivalent	10 (5%)	9 (6%)
Junior college graduate or some college or technical school	24 (13%)	28 (19%)
College graduate	73 (40%)	50 (35%)
Any postgraduate schooling	75 (41%)	57 (40%)
Parity:		
0	80 (44%)	64 (44%)
1	69 (38%)	57 (39%)
> 1	34 (19%)	25 (17%)
Smoking pregnancy (no/yes)	172 (92%) / 15(8%)	132 (90%)/15 (10%)
Marital status		
Married	166 (91%)	135 (94%)
Single	13 (7%)	7 (5%)
Divorced	3 (2%)	2 (1%)
Infants (boys/girls)	85 (45%) / 102 (55%)	82 (56%)/65 (44%)

Continuous values are reported as median (minimum, interquartile range: Q1–Q3, maximum), and categorical values as relative and absolute frequencies and relative frequencies

# Study population subgroup 1 with essential and non-essential element concentrations data available in paired urine samples collected at 6 weeks and 1 year of age. Maternal education contains 5 missing values. Parity contains 4 missing values. Marital status contains 5 missing values

* Study population subgroup 2 with essential and non-essential element concentrations and rice consumption at 1 year of age. Maternal BMI contains 1 missing value. Maternal education contains 3 missing values. Parity contains 1 missing value. Marital status contains 3 missing values

## Data Availability

Analytic data used in this study are included in the manuscript figures and tables and its Supplementary Information files.
