# Supplementary material for: Dietary Exposure to Essential and Non-essential Elements During Infants’ First Year of Life in the New Hampshire Birth Cohort Study
Source: Expo Health. Author manuscript; Available in PMC 2023 Mar 1. (PMC9971144; doi:10.1007/s12403-022-00489-x)
Supplement: Supplemental Material [file NIHMS1817760-supplement-Supplemental_Material.docx]

# Supplemental information

## Table S1. Limit of detection (LOD) and imputed values in paired urine samples at 6 weeks and 1 year of age.

| 6 weeks of age |  |  |  |  | 1 year of age |  |  |
| --- | --- | --- | --- | --- | --- | --- | --- |
| Metal | LOD (µg/L) | n (%) < LOD | n (%) imputed values^x^ |  | LOD (µg/L) | *n* (%) < LOD | *n* (%) imputed values^x^ |
| As | 0.01 | 1 (0.5) | 0 |  | 0.02 | 0 | 0 |
| Al | 3.70 | 0 | 0 |  | 4.20 | 0 | 0 |
| Cd | 0.03 | 9 (4.8) | 0 |  | 0.04 | 6 (3.2) | 0 |
| Co | 0.02 | 0 | 0 |  | 0.02 | 0 | 0 |
| Cr | 0.30 | 113 (60.4) | 20 (10.7) |  | 0.30 | 62 (33.1) | 5 (2.6) |
| Cu | 0.60 | 0 | 0 |  | 0.80 | 0 | 0 |
| Fe | 8.00 | 1 (0.5) | 0 |  | 6.00 | 2 (1.0) | 0 |
| Hg | 0.30 | 151 (80.7) | 73 (39.0) |  | 0.30 | 130 (69.5) | 18 (9.6) |
| Mn | 0.20 | 0 | 0 |  | 0.20 | 0 | 0 |
| Mo | 0.10 | 1 (0.5) | 0 |  | 0.10 | 0 | 0 |
| Ni | 0.30 | 0 | 0 |  | 0.30 | 0 | 0 |
| Pb | 0.03 | 0 | 0 |  | 0.04 | 0 | 0 |
| Sb | 0.03 | 0 | 0 |  | 0.04 | 1 (0.5) | 0 |
| Se | 0.80 | 0 | 0 |  | 0.80 | 0 | 0 |
| Sn | 0.40 | 12 (8.3) | 0 |  | 0.30 | 0 | 0 |
| U | 0.02 | 0 | 0 |  | 0.02 | 2 (1.0) | 0 |
| V | 0.09 | 73 (39.0) | 0 |  | 0.08 | 14 (7.4) | 0 |

*N* = 187. ^X^The value of LOD/√2 was imputed only when the ICP-MS standard calibration curve provided zero or negative values (Lubin et al., 2004). The Al and Sn urine concentrations at 6 weeks of age had 43 missing values each that were imputed using MICE (Buuren, 2011). The As concentrations refer to the sum of inorganic arsenic, monomethylarsonic acid, and dimethylarsinic acid.

## Table S2. Limit of detection (LOD) and imputed values in urine samples from infants of 1 year of age with data on rice consumption.

| Essential and non-essential elements | LOD (µg/L) | *n* (%) < LOD | *n* (%) imputed values^x^ |
| --- | --- | --- | --- |
| As | 0.10 | 0 | 0 |
| Cd | 0.02 | 0 | 0 |
| Co | 0.01 | 0 | 0 |
| Cr | 0.30 | 10 (6.8) | 0 |
| Cu | 0.15 | 0 | 0 |
| Fe | 3.00 | 0 | 0 |
| Mn | 0.05 | 0 | 0 |
| Mo | 0.02 | 0 | 0 |
| Ni | 0.15 | 0 | 0 |
| Pb | 0.01 | 1 (0.7) | 0 |
| Sb | 0.02 | 1 (0.7) | 0 |
| Se | 0.04 | 0 | 0 |
| U | 0.01 | 1 (0.7) | 0 |
| V | 0.10 | 64 (43.5) | 52 (35.3) |

*N* = 147. ^X^The value of LOD/√2 was imputed only when the ICP-MS standard calibration curve provided zero or negative values (Lubin et al., 2004). The As concentrations refer to the sum of inorganic arsenic, monomethylarsonic acid, and dimethylarsinic acid.

## Table S3: Essential and non-essential element concentrations (µg/L) in paired urine samples collected at 6 weeks and 1 year of age.

| Essential and non-essential elements | 6 weeks of age | 1 year of age | Median ratio ($\frac{1 year}{6 weeks}$) | t-statistic for paired t-test (df=186) | *P*-value |
| --- | --- | --- | --- | --- | --- |
|  |  |  |  |  |  |
| Al | 86.09 (9.48, 55.50 - 138.91, 543.88) | 113.66 (7.47, 71.16 – 187.88, 6191.81) | 1.3 | 2.58 | 0.011 |
| As | 0.20 (0.01, 0.06 - 0.33, 1.61) | 2.31 (0.10, 1.02 - 4.38, 18.97) | 14.8 | 12.74 | <0.001 |
| Cd | 0.12 (0.01, 0.08 - 0.20, 0.95) | 0.13 (0.01, 0.08 - 0.22, 5.85) | 1.1 | 2.00 | 0.046 |
| Hg | 0.14 (0.00, 0.04 - 0.23, 1.49) | 0.17 (0.01, 0.08 - 0.35, 1.35) | 1.4 | 2.71 | 0.007 |
| Pb | 0.55 (0.10, 0.30 - 0.80, 4.77) | 0.57 (0.07, 0.34 - 1.17, 9.66) | 1.3 | 2.55 | 0.012 |
| Sb | 1.82 (0.06, 0.94 - 4.84, 112.53) | 4.20 (0.04, 1.66 - 6.62, 35.08) | 1.6 | 2.03 | 0.044 |
| Sn | 1.25 (0.16, 0.89 - 2.21, 12.74) | 2.17 (0.53, 1.35 - 3.99, 33.69) | 1.7 | 4.15 | <0.001 |
| U | 1.20 (0.06, 0.87 - 2.07, 7.57) | 1.17 (0.00, 0.74 - 2.04, 9.20) | 1.0 | -0.82 | 0.416 |
| V | 0.11 (0.03, 0.08 - 0.14, 0.74) | 0.15 (0.02, 0.11 - 0.24, 5.08) | 1.5 | 3.41 | 0.001 |
| Co | 0.19 (0.04, 0.14 - 0.27, 1.54) | 0.39 (0.03, 0.26 - 0.57, 16.59) | 1.9 | 3.97 | <0.001 |
| Cr | 0.25 (0.01, 0.18 - 0.38, 28.30) | 0.40 (0.01, 0.23 - 0.68, 10.63) | 1.7 | 0.35 | 0.726 |
| Cu | 14.72 (2.48, 10.37 - 22.19, 124.02) | 12.76 (1.61, 8.37 - 19.37, 155.42) | 0.9 | -0.98 | 0.330 |
| Fe | 78.36 (6.53, 56.68 - 109.99, 409.72) | 89.82 (2.70, 63.00 - 137.65, 746.91) | 1.2 | 2.08 | 0.039 |
| Mn | 55.31 (3.40, 38.85 - 79.90, 278.69) | 53.21 (0.21, 27.18 - 96.63, 447.23) | 0.9 | 0.74 | 0.462 |
| Mo | 1.05 (0.07, 0.59 - 2.36, 16.43) | 45.36 (0.43, 20.04 - 76.15, 267.43) | 31.8 | 15.34 | <0.001 |
| Ni | 1.80 (0.15, 1.30 - 2.61, 13.08) | 3.29 (0.66, 2.23 - 4.93, 58.34) | 1.7 | 5.51 | <0.001 |
| Se | 14.14 (3.17, 9.39 - 21.40, 64.91) | 36.39 (5.60, 20.59 - 59.33, 183.15) | 2.2 | 10.93 | <0.001 |

*N* = 187. Reported values refer to median (minimum, interquartile range: Q1-Q3, maximum). The As concentrations refer to the sum of inorganic arsenic, monomethylarsonic acid, and dimethylarsinic acid.

## Table S4: Essential and non-essential element concentrations collected at 1 year of age from infants with available data on rice consumption at 1 year of age.

| Essential and non-essential elements | Rice consumers (*n* = 75) | No rice consumers (*n* = 72) | Ratio median concentrations ($\frac{rice consumers}{no rice consumers}$) | t-statistic for two-sample t-test | *P*-value |
| --- | --- | --- | --- | --- | --- |
|  |  |  |  |  |  |
| As | 2.96 (0.28, 1.63 - 6.78, 33.37) | 1.88 (0.24, 1.06 - 3.42, 14.48) | 1.6 | -3.43 | 0.001 |
| Cd | 0.26 (0.05, 0.160 - 0.38, 0.83) | 0.25 (0.05, 0.167 - 0.33, 0.99) | 1.1 | -1.57 | 0.118 |
| Pb | 0.84 (0.08, 0.362 - 1.53, 42.88) | 0.60 (0.07, 0.36 - 1.17, 5.21) | 1.4 | -1.33 | 0.187 |
| Sb | 0.78 (0.02, 0.31 - 2.41, 11.68) | 1.06 (0.13, 0.45 - 2.26, 20.77) | 0.7 | 0.55 | 0.586 |
| U | 1.13 (0.00, 0.664 - 1.99, 7.25) | 1.13 (0.13, 0.66 - 1.67, 4.48) | 1.0 | -0.73 | 0.469 |
| V | 0.18 (0.06, 0.07 - 0.33, 1.38) | 0.13 (0.02, 0.07 - 0.34, 0.66) | 1.4 | -1.34 | 0.181 |
| Co | 0.28 (0.08, 0.19 - 0.47, 2.59) | 0.26 (0.04, 0.187 - 0.42, 2.79) | 1.1 | -0.03 | 0.975 |
| Cr | 0.69 (0.22, 0.468 - 0.93, 3.46) | 0.66 (0.15, 0.47 - 0.95, 3.60) | 1.0 | -0.28 | 0.780 |
| Cu | 14.84 (1.19, 9.75 - 22.77, 68.44) | 12.58 (1.55, 8.63 - 16.53, 124.23) | 1.2 | -1.19 | 0.234 |
| Fe | 135.20 (20.62, 66.08 - 229.36, 697.27) | 113.77 (24.71, 73.45 - 179.61, 501.83) | 1.2 | -1.49 | 0.140 |
| Mn | 52.58 (0.26, 30.05 - 93.80, 204.73) | 56.51 (5.46, 22.15 - 83.79, 148.47) | 0.9 | -1.26 | 0.211 |
| Mo | 67.01 (6.05, 33.85 - 116.50, 562.96) | 45.90 (1.59, 18.78 - 82.66, 341.98) | 1.5 | -2.18 | 0.031 |
| Ni | 3.48 (0.54, 2.71 - 5.44, 23.06) | 2.96 (0.46, 1.90 - 4.51, 11.60) | 1.2 | -1.96 | 0.053 |
| Se | 45.86 (5.82, 23.84 - 80.58, 243.72) | 35.15 (4.44, 18.53 - 79.59, 182.93) | 1.3 | -0.84 | 0.402 |

*N* = 147. Reported values refer to median (minimum, interquartile range: Q1-Q3, maximum). The As concentrations refer to the sum of inorganic arsenic, monomethylarsonic acid, and dimethylarsinic acid.

## Figure S1: Flowchart for infants with paired urinary essential and non-essential elements at 6 weeks and 1 year of age (A), and infants with data on rice consumption and urinary essential and non-essential elements at 1 year of age (B).


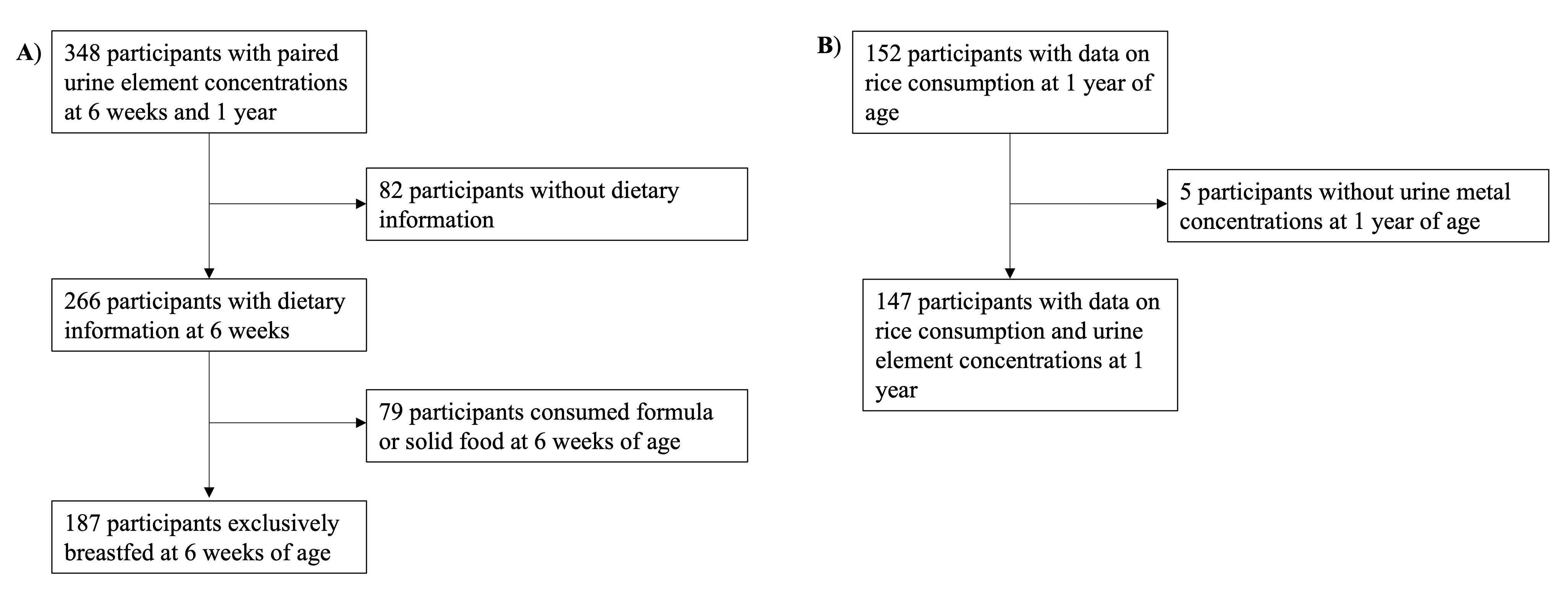


## Figure S2: Distributions of the $\frac{\boldsymbol{1 year}}{\boldsymbol{6 weeks}}$ of age natural-logarithm-transformed (Ln) urinary element concentrations.


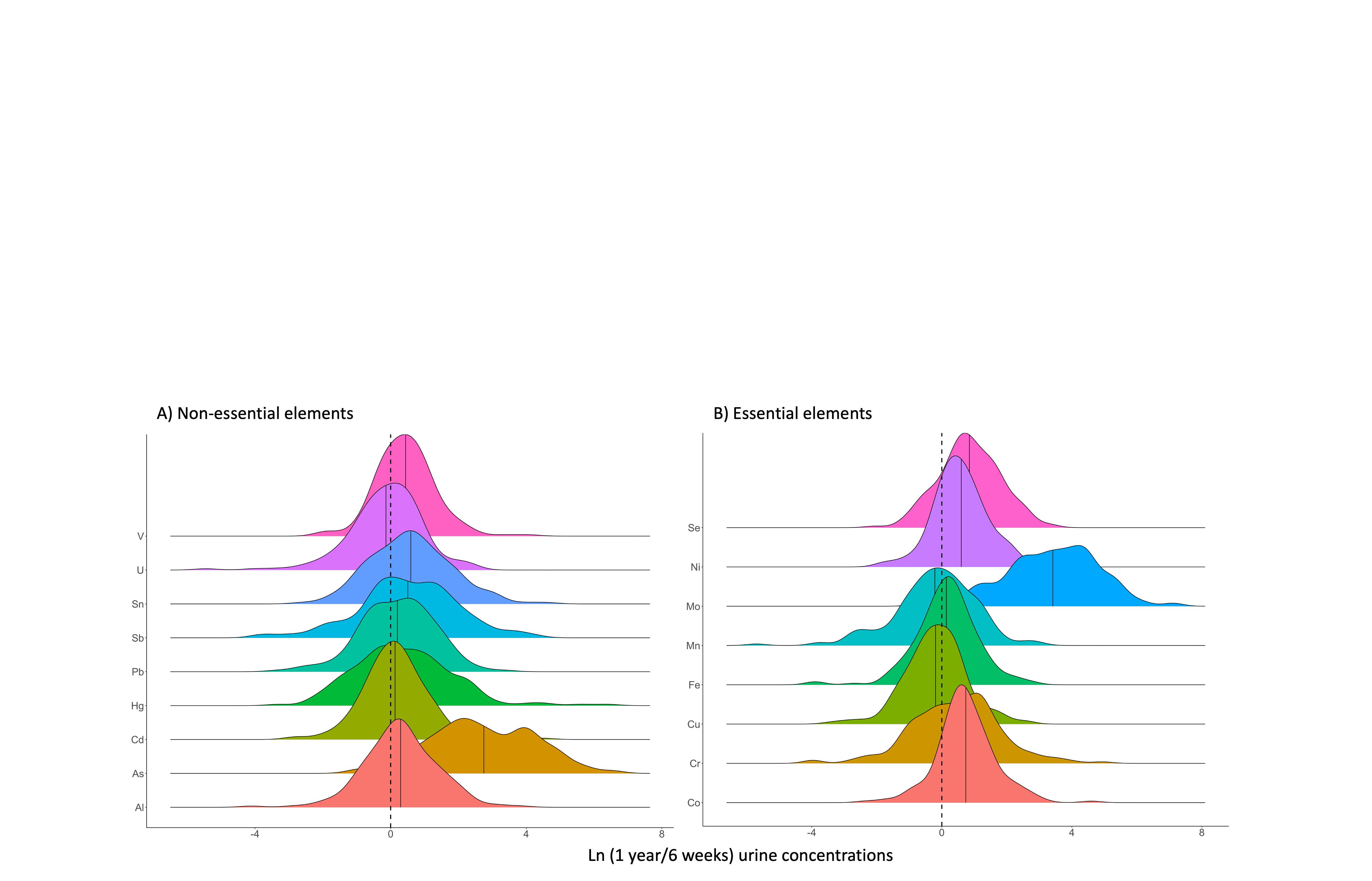


**A**) Non-essential. **B**) Essential elements. Dashed vertical black lines shows no change in the urinary element concentration at 6 weeks and 1 year of age (i.e., Ln ($\frac{1 year}{6 weeks}$ concentration = 0). The As concentrations refer to the sum of inorganic arsenic, monomethylarsonic acid, and dimethylarsinic acid.

## Figure S3. WQS model regression index (positive weights) for urine essential and non-essential element concentrations at transitioning from 6 weeks to 1 year of age.


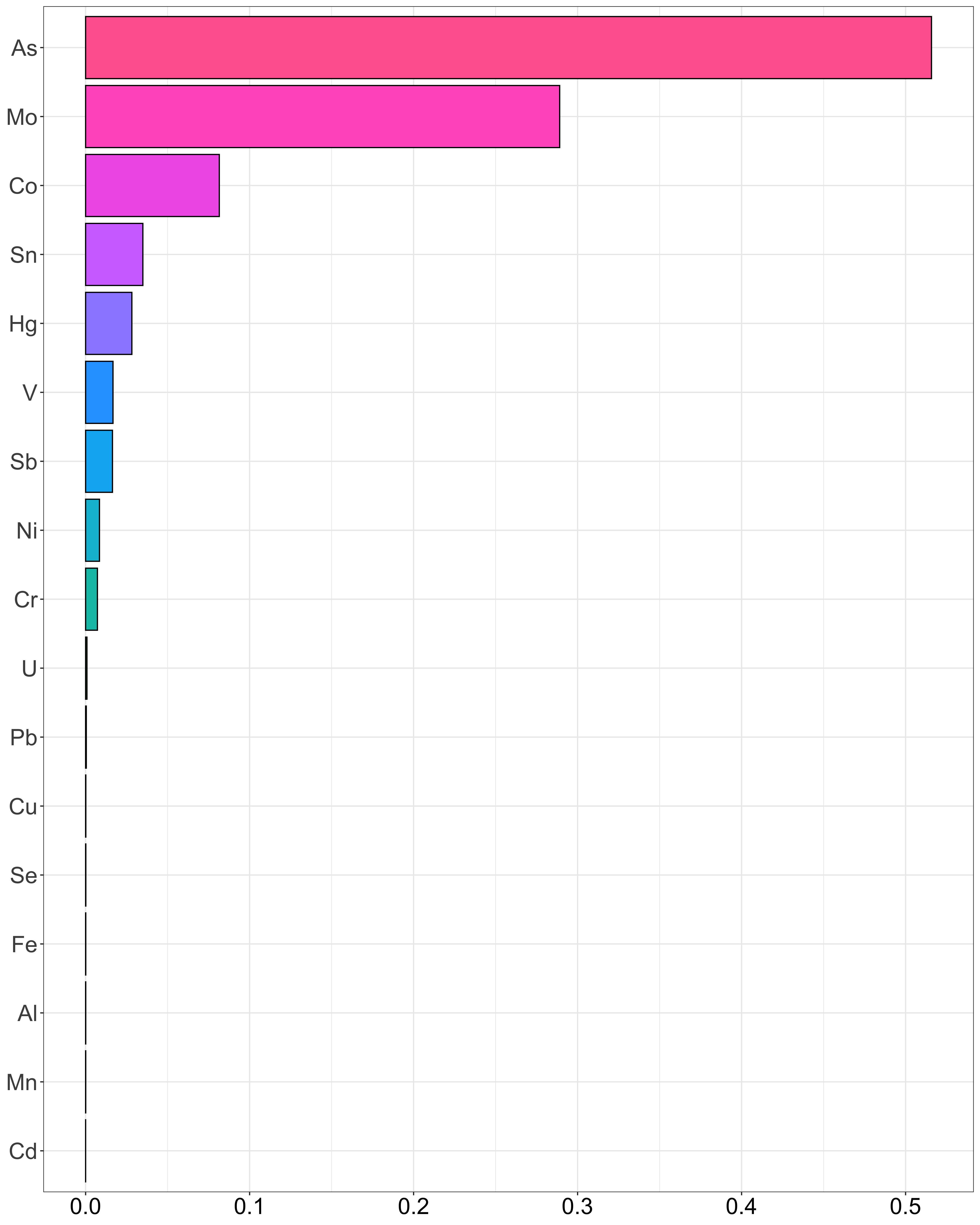


The mean weights are 0.5160000 (As), 0.2890000 (Mo), 0.0815000 (Co), 0.0349000 (Sn), 0.0282000 (Hg), 0.0167000 (V), 0.0164000 (Sb), 0.0084500 (Ni), 0.0072000 (Cr), 0.0008100 (U), 0.0004150 (Pb), 0.0001340 (Cu), 0.0001330 (Se), 0.0000984 (Fe), 0.0000838 (Al), 0.0000783 (Mn), and 0.0000088 (Cd). Estimate = 2.53, standard error = 0.437, z value = 5.79, and *p*-value <0.001. The As concentrations refer to the sum of inorganic arsenic, monomethylarsonic acid, and dimethylarsinic acid.

## Figure S4: Visualization of ln-transformed urinary concentrations (lower triangle) and Spearman’s correlation matrix (upper triangle) for each pair of essential and non-essential element concentrations in the infant urine samples collected at 6 weeks of age.


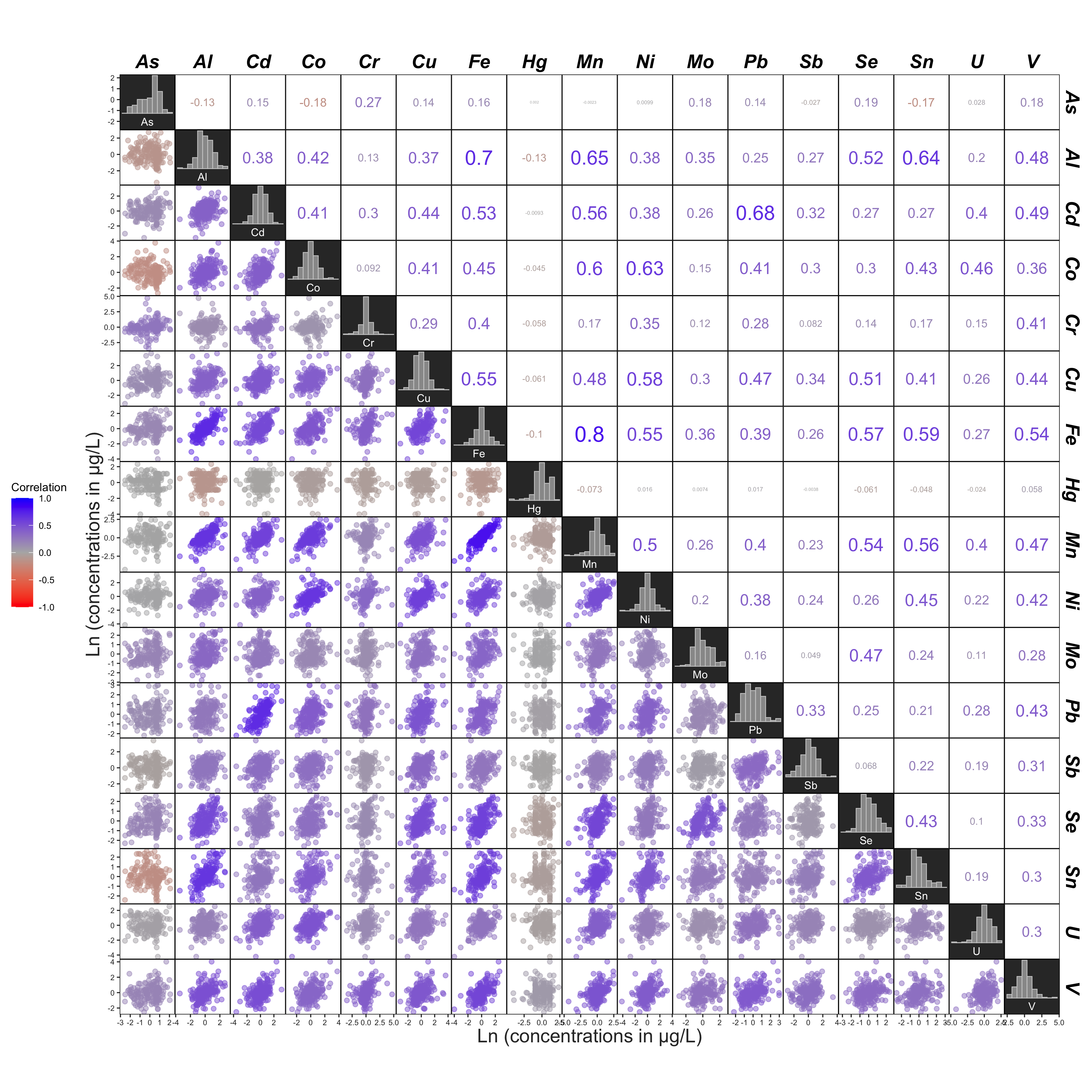


*N* = 187. The As concentrations refer to the sum of inorganic arsenic, monomethylarsonic acid, and dimethylarsinic acid. The color ranges from red to blue refers to the spearmans’ correlation coefficient from -1 to 1, respectively. The diagonal shows the distribution of the Ln (concentrations in µg/L) of each essential and non-essential element.

## Figure S5: Visualization of ln-transformed urinary concentrations (lower triangle) and Spearman’s correlation matrix (upper triangle) for each pair of essential and non-essential element concentrations in the infant urine samples collected at 1 year of age.


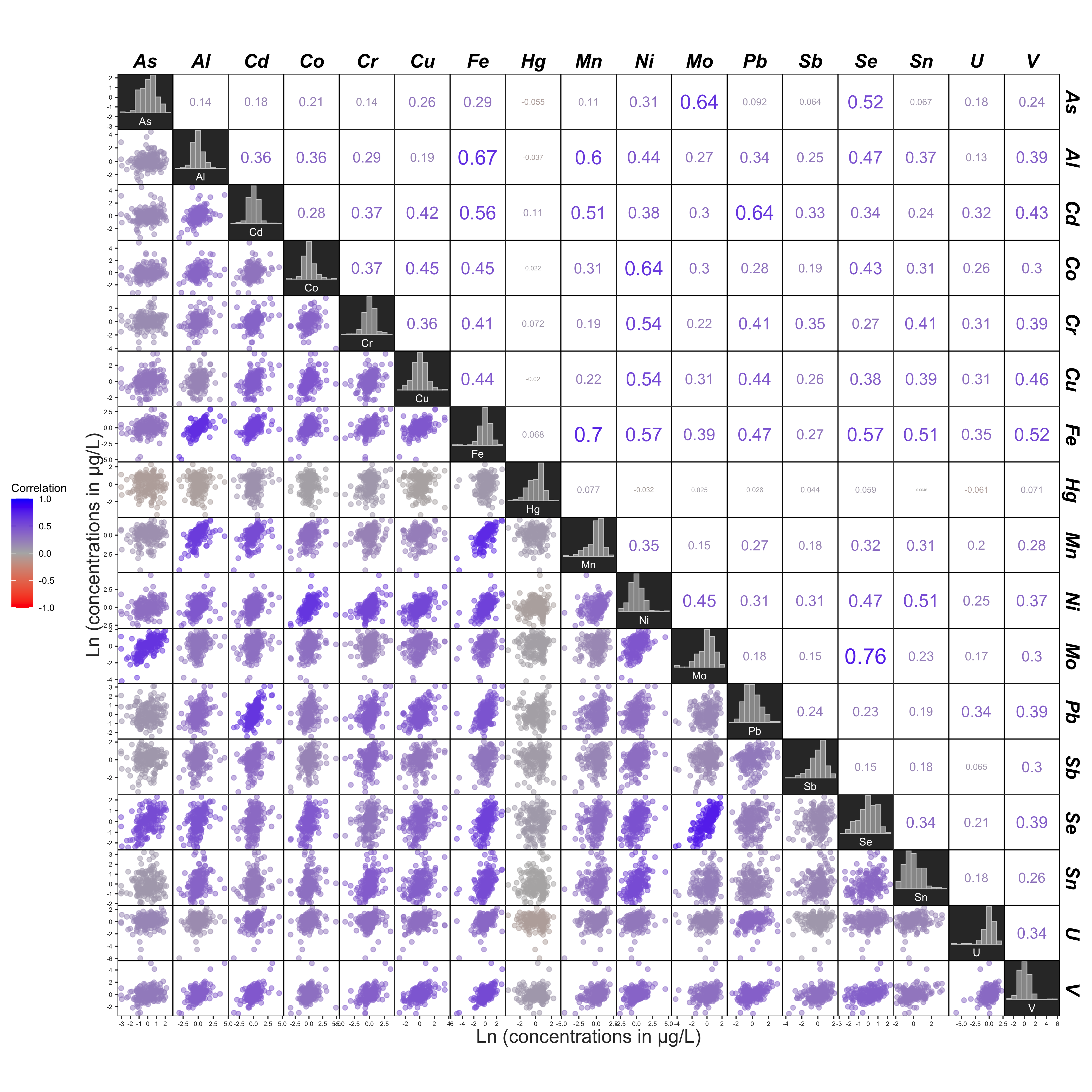


*N* = 187. The As concentrations refer to the sum of inorganic arsenic, monomethylarsonic acid, and dimethylarsinic acid. The color ranges from red to blue refers to the spearmans’ correlation coefficient from -1 to 1, respectively. The diagonal shows the distribution of the Ln (concentrations in µg/L) of each essential and non-essential element.

## Figure S6: Visualization of ln-transformed urinary concentrations (lower triangle) and Spearman’s correlation matrix (upper triangle) for essential and non-essential element concentrations in urine samples collected at 1 year of age from rice-consumer infants.


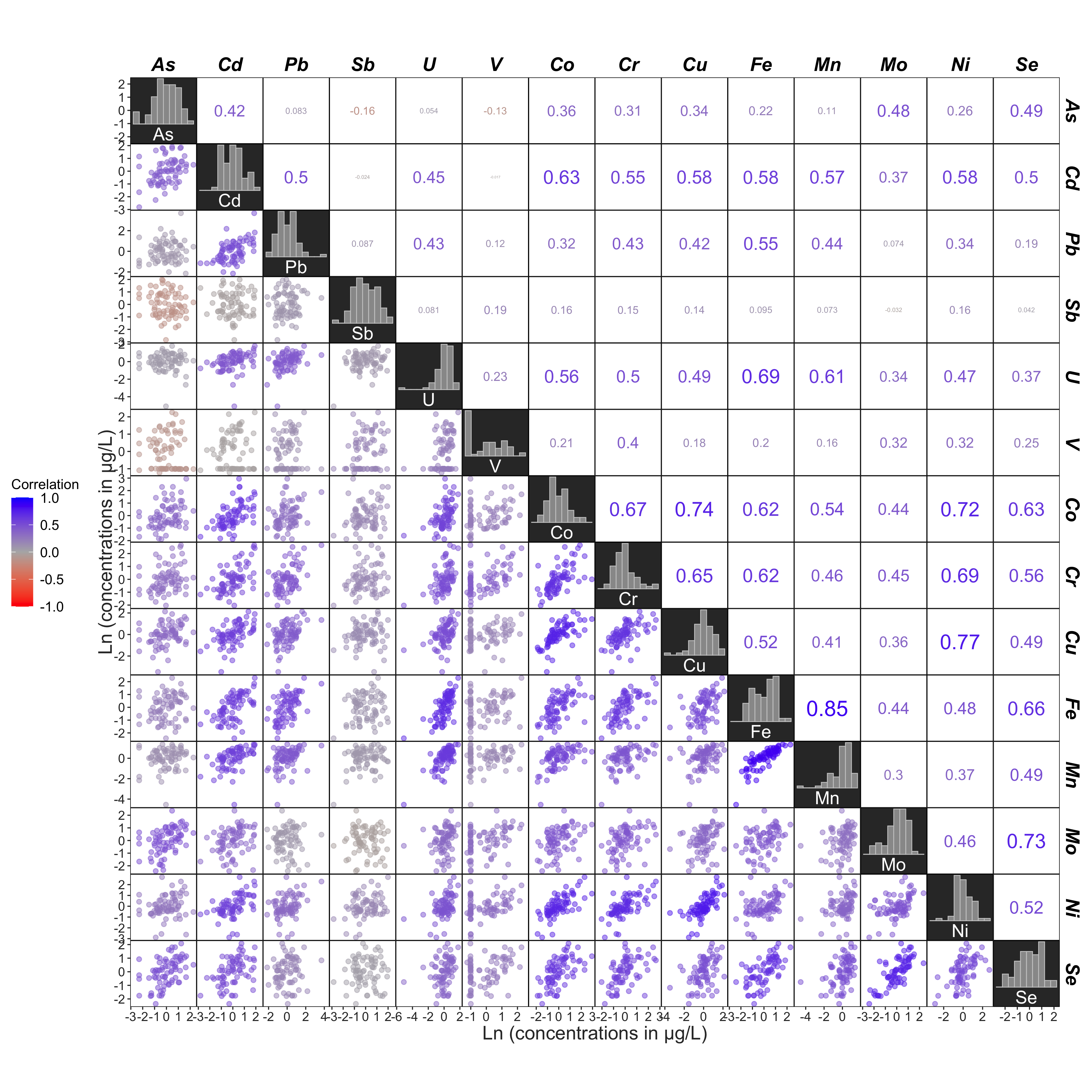


*N* = 75. The As concentrations refer to the sum of inorganic arsenic, monomethylarsonic acid, and dimethylarsinic acid. The color ranges from red to blue refers to the spearmans’ correlation coefficient from -1 to 1, respectively. The diagonal shows the distribution of the Ln (concentrations in µg/L) of each essential and non-essential element.

## Figure S7: Visualization of ln-transformed urinary concentrations (lower triangle) and Spearman’s correlation matrix (upper triangle) for essential and non-essential element concentrations in urine samples collected at 1 year of age from non-rice-consumer infants.


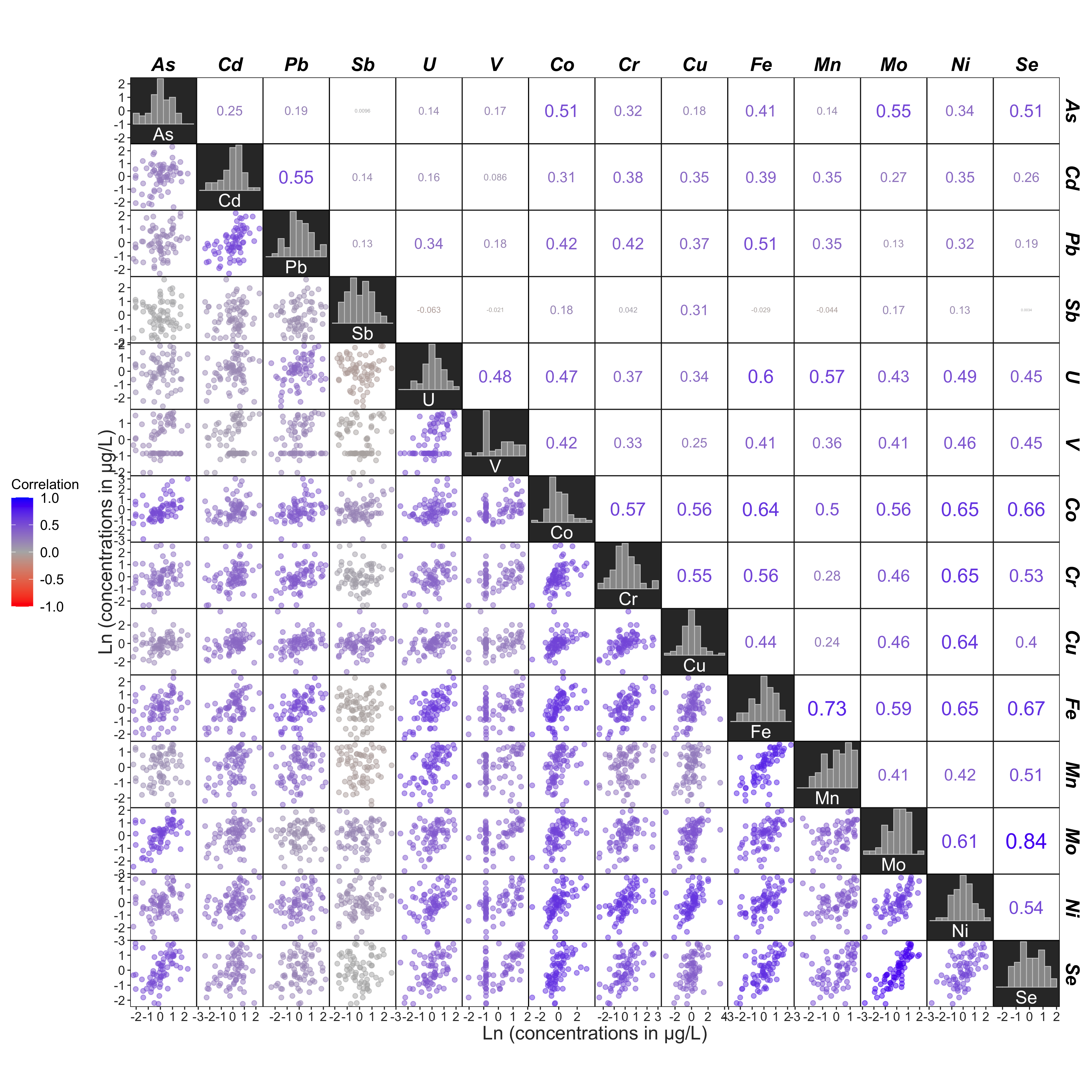


*N* = 72. The As concentrations refer to the sum of inorganic arsenic, monomethylarsonic acid, and dimethylarsinic acid. The color ranges from red to blue refers to the spearmans’ correlation coefficient from -1 to 1, respectively. The diagonal shows the distribution of the Ln (concentrations in µg/L) of each essential and non-essential element.
